# Comparative Transcriptome Analysis of *Rhynchophorus ferrugineus* (Coleoptera: Curculionidae) Reveals Potential Mechanisms Involved in the Toxication and Detoxification of the External Immune Compound p-Benzoquinone Present in Oral Secretions

**DOI:** 10.3390/insects16101044

**Published:** 2025-10-11

**Authors:** Juan Chen, Yu-Chen Pu, Wen-Qing You, Ya-Nan Ji, Can-Hui Ding, Zong-Wei Zheng, Yi-Fan Wang, You-Ming Hou

**Affiliations:** 1 Engineering Technological Center of Mushroom Industry, School of Biological Science and Biotechnology, Minnan Normal University, Zhangzhou 363000, China; 19959497098@163.com; 2 Key Laboratory of Landscape Plants with Fujian and Taiwan Characteristics of Fujian Colleges and Universities, School of Biological Science and Biotechnology, Minnan Normal University, Zhangzhou 363000, China; youwenqing02@163.com (W.-Q.Y.); 12302003023@fafu.edu.cn (C.-H.D.); 17750357920@163.com (Z.-W.Z.); wyf3121129612@163.com (Y.-F.W.); 3 State Key Laboratory of Agricultural and Forestry Biosecurity, Fujian Agriculture and Forestry University, Fuzhou 350002, China; yananji1@outlook.com; 4 Fujian Provincial Key Laboratory of Insect Ecology, College of Plant Protection, Fujian Agriculture and Forestry University, Fuzhou 350002, China

**Keywords:** red palm weevil, benzoquinone, oral secretions, external immune defense, metabolic detoxifying enzymes, transcriptome

## Abstract

**Simple Summary:**

The red palm weevil is a devastating invasive pest that damages palm trees. The oral cavity of larvae can secrete an external immune-active compound called p-benzoquinone to defend against pathogens. However, p-benzoquinone is also toxic to certain organisms. This study investigated how p-benzoquinone affected the larvae and how the larvae detoxified p-benzoquinone. We found that p-benzoquinone disrupted the larval epidermis by regulating the expression of genes involved in digestion and pigmentation, such as chitinase and phenoloxidase genes, leading to abnormal molting or cuticular melanization. Meanwhile, the larvae counteracted the toxicity of p-benzoquinone by activating detoxifying enzymes, including cytochrome P450, glutathione S-transferase and ATP-binding cassette transporter. Understanding these potential mechanisms will help to develop new pest control methods by targeting the weevil’s natural detoxification system, offering a safer and more effective way to protect palm trees.

**Abstract:**

p-Benzoquinone (PBQ), a highly toxic compound, is the main active component in larval oral secretions of red palm weevil (RPW), *Rhynchophorus ferrugineus*, playing critical roles in external immunity and pathogen defense. In this study, we demonstrated that pathogens effectively induce RPW larval external immune responses. On this basis, the toxicity of PBQ to third-instar larvae was determined, with poisoning symptoms observed. The differences in gene expression between larvae before and after treatment with PBQ were analyzed by transcriptome sequencing to potentially involve the mechanisms of PBQ toxicity on larvae and the mechanisms of detoxification in the infected larvae. The results indicated that PBQ exposure was associated with altered expression of chitinase (CHI) and phenoloxidase (PO) genes in RPW larvae, which not only affects the digestion and degradation of the old cuticle but also activates phenoloxidase, further oxidizing tyrosine for its conversion into DOPA and dopamine, resulting in the generation of melanin and different degrees of cuticular melanization. The transcriptional changes further suggest that RPW larvae may employ metabolic processes to counteract the external immune-active compound PBQ toxicity by regulating the expression levels of detoxifying enzyme-encoding genes, such as cytochrome P450 (*CYP450*), glutathione S-transferase (*GST*), and ATP-binding cassette transporter (*ABC*). Our research provides potential novel strategies for pest control by targeting insect metabolic detoxification systems.

## 1. Introduction

Insects rely on a unique innate immune system complemented by the gut microbiota, which helps defend against pathogens [1,2]. However, an increasing number of studies indicate that the immune defense of insects against pathogens begins with specific physical and chemical barriers outside the body, such as the hard exoskeleton, chitinous tracheae, the peritrophic membrane matrix of the gut, and defensive secretions that effectively inhibit the growth of microorganisms in vitro [3,4,5]. In fact, these external immune defense mechanisms, particularly chemical secretions, enable the early mitigation of potential pathogenic threats [6,7].

Currently, the external secretions of beetles, which primarily include quinones, aldehydes, short-chain organic acids, aromatics, and certain beetle-specific compounds, have been extensively studied [8,9,10]. The composition and quantity of secretions are influenced by both genetic and environmental factors, including sex, developmental stage, reproductive status, diet, season, living environment, and defensive targets. The chemical defensive secretions of *Oreina gloriosa* contain at least 16 components, approximately half of which are genetically determined [11]. In *Romalea guttata* and *Taeniopoda eques*, elder instar larvae release more secretions than young larvae do, female adults release more secretions than male adults do, heavier individuals release larger quantities of secretions than lighter individuals do, and individuals in the reproductive period produce more secretions, which are also more complex in composition [12].

Quinones and their derivatives, especially benzoquinone, are major nonspecific toxic chemical defense elements present in the external secretions of many Coleoptera insects and are active components of external immunity [13]. These compounds can not only inhibit the growth of pathogens such as bacteria and fungi in vitro but also exhibit repellent or stimulatory effects on predators [8,14,15]. p-Benzoquinone (PBQ) is an unsaturated cyclic diketone that is highly toxic to organisms. Mofty et al. [16] investigated the toxicity of quinone secretions by feeding *Swiss albino* mice with beetle secretions containing PBQ and cookies made from flour containing PBQ secretions. The results indicated that quinone secretions can induce tumor formation. Moreover, Chambers and Rowan [17] reported that PBQ can cause acute intoxication syndrome in cockroaches.

The biological activity of most quinone compounds is influenced by their redox properties [18]. Therefore, the propensity for biological and chemical redox reactions is a significant property of quinones. Quinones form corresponding diphenolic compounds through biological reduction. When these diphenols encounter chemical or biological oxidants, they can be reoxidized back into quinones. PBQ is widely present in plants and animals in nature, and its cytotoxicity is one of its important biological properties. Research has shown that PBQ can react with proteins and DNA to form reactive oxygen species (ROS), thereby damaging cells [19,20]. Additionally, PBQ can alter chromosomal structures within cells, as evidenced by different chromosomal aberrations observed in human leukemia cells and mouse bone marrow cells upon pulse treatment with 2,6-dimethyl-PBQ from arachnid secretions [21]. Furthermore, PBQ can cause cellular damage to the nervous system of organisms, such as by inhibiting the transport channels of cations, leading to depolarization of the postsynaptic membrane of the sixth abdominal segment of cockroaches [17]. In addition to causing cytotoxicity, PBQ can also induce genotoxicity by disrupting topoisomerase IIIα (TOPOIIIα), an enzyme essential for DNA repair. After treatment with PBQ for 24 h, an increase in the concentration of double-strand DNA break markers was detected [22].

Several studies have shown that defensive secretions may also have certain toxic side effects on the secreting organism. The confused flour beetle (*Tribolium confusum*) secretes a liquid with a pungent odor containing a mixture of compounds such as methyl-PBQ and ethyl-PBQ when stimulated. This secretion significantly affects the physiology of individuals, causing larvae and pupae to deform and develop into adults with malformations [23,24]. Yezerski et al. [25] also reported that benzoquinones effectively killed *T. confusum* in the absence of a flour medium. Genetic and environmental factors determine the secretory volume of defensive chemicals [25]. This kind of secretion functions as an external defense factor, providing immune protection to the host. However, at high concentrations, it is toxic to the secreting individual [8]. Consequently, natural selection may optimize the secretory levels of external immune compounds in individuals under normal conditions. It remains to be further explored whether beetles can effectively regulate the secretion of antimicrobial substances to avoid poisoning. If beetles can indeed strategically adjust the secretion of quinones, their environmental adaptability can be increased.

Gokhale et al. [26] hypothesized that the production of external secretions by beetles has both a maximum threshold and a minimum threshold: if external secretion production exceeds the maximum threshold, it may have potential adverse effects on both the individual and its offspring, thereby reducing their fitness and adaptability; if it falls below the minimum threshold, the antimicrobial function of quinones is suppressed even when they are adequately diffused into the environment. Therefore, beetles must possess necessary protective mechanisms to counteract the toxic effects of defensive quinones present in the secretions of their external immune system. Previous studies have reported various mechanisms by which insects reduce or avoid self-intoxication caused by their own toxic compounds produced for defensive purposes [27]. For example, Tenebrionidae beetles effectively prevent harm from their own toxic secretions through the use of both internal and external cuticular tissues [27]. *Tribolium* beetles use quinones as tanning agents to harden the cuticle [28]. The red flour beetle (*Tribolium castaneum*) stores secretions produced by internal cellular organelles within storage vesicles formed by cuticle invagination [29]. Similar to beetles, other insects have evolved analogous defense system. Senior *Neocapritermes taracua* worker ants excel in group defense and possess a two-component activation defense system composed of two separately stored secretions. In times of danger from intruders, these workers sacrifice themselves to ensure the safety of the nest by rupturing their bodies, causing the components to mix and react, producing a toxic, sticky benzoquinone mixture harmful to enemies [30]. *Mastotermes darwiniensis* may employ a protective system mediated by oxidoreductases, such as glucose dehydrogenase, to reduce oxidized benzoquinone back to hydroquinone at the synthesis site [31]. Once this self-protection system is disrupted, the pest is inevitably harmed by its own secretions. Therefore, insects must possess well-developed detoxification systems and mechanisms.

The red palm weevil (RPW), *Rhynchophorus ferrugineus* (Coleoptera: Curculionidae), is a highly destructive invasive pest that severely impacts palm plants, causing significant economic losses to urban garden landscapes and the palm industry [32]. Previous studies have shown that when RPW larvae are subjected to immune challenge or mechanical stimulation, they secrete a brown-colored liquid from their oral cavity that significantly inhibits the growth of various microorganisms in vitro [33], thereby severely weakening the biological control efficacy of pathogens. PBQ is the primary active compound in the larval oral secretions responsible for external immune function in this species [33]. In particular, the arylsulfatase B (*ARSB*) gene is involved in the regulation of the biosynthesis and metabolism of PBQ, maintaining its concentration within the tolerance range of the individual [10]. In addition to regulating the secretory volume of PBQ, it is crucial for RPW to effectively detoxify this compound to avoid its toxic effects from external immune substances on individuals. However, this mechanism remains unclear.

We hypothesized that the hypothesis that exposure to PBQ in oral secretions causes dose-dependent toxic phenotypes (abnormal molting, cuticular melanization, and mortality) in third-instar RPW larvae, and that acute PBQ exposure elicits a coordinated detoxification and melanization response—specifically, rapid induction and increased activity of cytochrome P450s (CYPs), glutathione-S-transferases (GSTs), ABC transporters (ABC), phenoloxidase (PO) and chitinases (CHI)—which causally contributes to PBQ tolerance. Here, we report the outcomes of experiments designed to test our hypothesis.

In this study, on the basis of our investigation of the impact of pathogen stress on the external immune defense of RPW larvae, we used Illumina RNA-seq to analyze the toxic effects of the defensive compound PBQ in the oral secretions of individuals and further elucidate potential detoxification mechanisms of the insect against PBQ. The present study aims to provide new approaches for pest control in the field by disrupting the metabolic detoxification system.

## 2. Materials and Methods

### 2.1. Insect Collection and Rearing

The RPW samples used in this study originated from a population collected in 2021 at Minnan Normal University, Zhangzhou City, Fujian Province, China (117.63° E, 24.51° N). After daily collection from the trap, the adults were brought back to the laboratory for breeding and population maintenance. The adults were paired and fed fresh sugarcane stems, which were replaced every 7 d. Once the eggs were successfully laid, they were carefully picked up with a brush and placed in Petri dishes with presoaked cotton wool at the bottom for incubation. The eggs hatched into larvae after 3 to 5 d of incubation. The newly hatched larvae were then transferred to clean Petri dishes and fed individually with fresh sugarcane stems, which were also replaced every 7 d until pupation. The pupae underwent metamorphosis into adults within 7 to 14 d. Both the population and the experimental insects were reared in a controlled environment chamber (Kesheng Experimental Instrument Co., Ltd., Ningbo, China) at 28 °C, with a relative humidity of 75%. However, the adults were reared under a 12 L:12 D light cycle, while the insects at all other stages were raised in full darkness to simulate their natural development inside palm trunks.

### 2.2. Preparation of Pathogen Suspensions

The pathogen used in this study was *Metarhizium anisopliae* (accession no. MF467274 in GenBank), which was isolated from diseased stiff RPWs as described by Pu et al. [34]. The *M. anisopliae* strain was inoculated onto potato dextrose agar (PDA; Shanghai Bioway Technology Co., Ltd., Shanghai, China) medium plates and incubated in a biochemical incubator at 25 °C for 7 d to produce many dark green conidia. The conidia were subsequently scraped into a 0.1% Tween-80 solution with sterile surgical blades on an ultraclean workbench. A pipette was then used to thoroughly suspend the fungal spores, and a syringe filled with sterile cotton wool was used to filter out the hyphae, thereby preparing a spore suspension. To determine the conidial concentration, 10 μL of the spore suspension was added to a hemocytometer and counted under a microscope. Sterile water was used to dilute the suspension to concentrations of 1.0 × 10^5^ conidia/mL and 1.0 × 10^7^ conidia/mL.

### 2.3. In Vitro Infection of RPW Larvae by Pathogens

A piece of filter paper was laid at the bottom of the Petri dish, and 1 mL of *M. anisopliae* conidial suspension at a sublethal concentration of 1.0 × 10^5^ conidia/mL was evenly dropped onto the filter paper. Sugarcane slices coated with 2 mL of the suspension at the concentration outlined above were placed into each Petri dish. To achieve immune challenge in vitro, one third-instar or seventh-instar larva that had been starved for 24 h was placed into each dish, and *M. anisopliae* was added and incubated for 12 h, 24 h or 48 h to infect the larvae. For the control group, the same procedure was carried out with 0.1% Tween-80.

### 2.4. Effects of Pathogen Stress on the External Immune Defense of RPW Larvae

#### 2.4.1. Collection of Oral Secretions

To collect oral secretions, the method described by Chen et al. [35] and Pu et al. [10] was followed. On an ultraclean workbench, the larva suffering from external pathogen stress was gently fixed between the fingers and thumb and softly touched by a 0.1–10 μL pipette tip at the mouth cavity. Usually, larvae are prompted to spit out oral secretions under such extrusion stimuli. The secretion sample from each individual larva was then collected into a 1.5 mL sterile Eppendorf tube. The tubes were labeled and quickly frozen in liquid nitrogen for 5 s before being transferred to −80 °C for storage. The secretions collected from each individual larva were used directly to measure secretion weight. In addition, to meet the minimal volume requirements (100 mL) for the assays, the secretions from 10 larvae were pooled for subsequent PBQ quantification and antimicrobial assays.

#### 2.4.2. Measurement of Secretion Levels

Each RPW larva was subjected to physical mechanical stimulation to release oral secretions, with a consistent level of stimulation intensity maintained. The centrifuge tube was weighed after the addition of the secretions, and the difference in weight from the empty tube was used to measure the quantity and efficiency of the secretions produced by the mouth cavity. The oral secretions from each individual larva were measured and analyzed, and 40 larvae were determined per treatment group (*n* = 40).

#### 2.4.3. Quantitative Determination of PBQ in Oral Secretions

To quantify the main external immune-active component PBQ, oral secretion samples were submitted to Shanghai Bioclouds Biological Technology Co., Ltd. (Shanghai, China) for targeted metabonomic assessment by gas chromatography-mass spectrometry (GC-MS). Oral secretions from 10 larvae were pooled as one biological replicate. Three independent biological replicates were performed per treatment group (*n* = 3). Authentic standard solutions (0.1 mg/mL PBQ diluted with methanol) were prepared, and a five-point calibration was performed by GC-MS. On the basis of the standard curve, the areas of the abundances from GC-MS were transformed to masses. The Samples were run on a 7980A gas chromatograph coupled to a 5975C mass spectrometer (Agilent, Palo Alto, CA, USA). Derivatized extracts of 1 μL were injected onto a nonpolar DB-5MS capillary column (30 m × 250 μm I.D., J&W Scientific, Folsom, CA, USA) with a G6500 CTC PAL autosampler (Agilent), and the injection was run in pulsed splitless mode. The procedures and conditions for GC-MS were as follows.

The injection port temperature of the chromatography instrument was set at 280 °C, with high-purity helium used as the carrier gas at a flow rate of 6.0 mL/min. The temperature program started at 60 °C, initially increased to 125 °C at a rate of 8 °C/min and then increased to 190 °C at a rate of 10 °C/min, 210 °C at a rate of 4 °C/min, and finally to 310 °C at a rate of 20 °C/min, after which this temperature was maintained for 8.5 min.

The ion source for mass spectrometry used electron impact ionization (EI) with an electron energy of 70 eV, an ion source temperature of 230 °C, and a quadrupole temperature of 150 °C. The scanning mode was set to full scan mode (SCAN), with a mass scanning range of *m*/*z* 50–600. Continuous sample analysis was conducted in a randomized sequence to mitigate the effects of instrument signal fluctuations.

#### 2.4.4. Antimicrobial Efficacy Assay

An experiment was conducted to assess the inhibitory effect of oral secretions from third-instar larvae on *M. anisopliae* after external infection, and the spore germination rate was determined to evaluate this efficacy. After the larvae were infected with a *M. anisopliae* spore suspension of 1.0 × 10^5^ conidia/mL, the collected oral secretions were diluted twofold with sterile water, and 15 µL was added to the grooves of a double concave slide. Then, 15 µL of a 1.0 × 10^7^ conidia/mL *M. anisopliae* spore suspension was added, followed by thorough mixing. The double concave slide was placed in a wet box with sterile water at the bottom, and the box was placed in a biochemical incubator at 25 °C for 9 h. Subsequently, the germination status of conidia from both the treatment group and the control group was observed under an optical microscope, and the spore germination rate was calculated. This rate was calculated by the formula *R* = *M*/*N* × 100%, where *R* represents the spore germination rate, *M* denotes the number of germinated spores, and *N* indicates the total number of spores counted. Spores were considered to have germinated when the length of the germ tube was greater than half of the spore radius. Each biological replicate consisted of oral secretions pooled from 10 larvae, and six independent biological replicates were performed per treatment group (*n* = 6).

### 2.5. Evaluation of the Biological Activity of the External Immune Compound PBQ on RPW Larvae

The PBQ standard (Macklin Biochemical Technology Co., Ltd., Shanghai, China) was diluted with sterile water to prepare a 9.500 mg/mL solution. It was then serially diluted in a 1.15-fold gradient to obtain concentrations of 8.261 mg/mL, 7.183 mg/mL, 6.246 mg/mL, and 5.432 mg/mL. Sugarcane slices were immersed separately in the aforementioned concentrations of PBQ solution for 30 min, followed by air-drying for 15 min. Three slices of sugarcane soaked in solution were placed into each Petri dish, into which one third-instar larva that had been starved for 24 h was placed. The sugarcane slices were replaced every 5 d to ensure consistent PBQ exposure and avoid degradation of the compound over time. This replacement schedule was determined on the basis of preliminary stability tests showing significant PBQ degradation and complete uptake by insects after 5 d under standard rearing conditions. The number of dead larvae was recorded daily for 10 d. Each treatment was performed in triplicate, with a total of 30 larvae. Distilled water was used as a control. The larval mortality in the bioassays was Abbott-corrected on the basis of the mortality in the control group [36]. The virulence parameters of PBQ in RPW larvae, including the sublethal concentration (LC_10_) and median lethal concentration (LC_50_), were further obtained through toxicity regression analysis.

### 2.6. Screening of Key Regulatory Genes from RPW Larvae Involved in the Toxication and Detoxification of PBQ

#### 2.6.1. Extraction of Total RNA

Third-instar RPW larvae were fed a PBQ solution at the LC_50_, and distilled water was used as a control, as described above. To elucidate the molecular mechanisms underlying tolerance to chronic PBQ exposure, we selected uniformly sized live specimens after 10 d of treatment and placed them in 1.5 mL centrifuge tubes with one specimen per tube. These specimens were immediately treated with liquid nitrogen at −196 °C for 10 min and then transferred to −80 °C for storage. Each treatment was performed on three biological replicates, with each replicate consisting of three larvae. Total RNA from the RPW larvae was extracted by the TRIzol method. The concentration, purity, and integrity of the RNA were assessed via 1% non-denaturing agarose gel electrophoresis and a biological analyzer. The specific procedures were carried out in accordance with methods described by Yang et al. [37].

#### 2.6.2. Transcriptome Sequencing

After extraction of total RNA from third-instar RPW larvae treated with PBQ in the treatment group and distilled water in the control group, cDNA libraries were constructed following the procedure described by Shen et al. [38]. After the library was qualified, high-throughput sequencing was performed with the Illumina HiSeq^TM^ 4000 platform by Guangzhou Gene Denovo Biotechnology Co., Ltd. (Guangzhou, China) for transcriptomic detection. The raw reads were deposited in the NCBI Sequence Read Archive (SRA) under the BioProject accession number PRJNA1333853.

Once transcriptome data were obtained, bioinformatics analysis was subsequently performed. We used fastp for quality control of the raw reads and filtered out low-quality data according to the following criteria: (1) reads containing adapters; (2) reads with a proportion of N bases greater than 10%; (3) reads consisting entirely of A bases; and (4) low-quality reads where the number of bases with a quality value Q ≤ 20 accounted for more than 50% of the entire read. The clean reads were then assembled with Trinity software (version 2.8.5) to acquire transcript sequences for subsequent analysis. These transcripts were then clustered using the TIGR Gene Indices clustering tools to reduce sequence redundancy, resulting in a non-redundant set of unique gene sequences (unigenes). A longer unigene N50 with fewer numbers indicates better assembly quality. The longest transcript from each gene was selected as the unigene for further analysis. To obtain more comprehensive information on gene function, sample data were annotated in gene functions across four major nucleotide and protein databases, including the Nr, KOG/COG, Swiss-Prot, and KEGG databases, by BLAST (version 2.6.0) comparison. Key differentially expressed genes (DEGs) and their metabolic pathway enrichment were screened and analyzed. The significance threshold of DEGs identification was selected as |log_2_(FC)| > 1 and FDR < 0.05 (as determined by DESeq2 tool), indicating that these genes may play crucial roles in the toxication and detoxification metabolism of PBQ by this insect.

Notably, by selecting surviving larvae after 10 d of LC_50_ exposure, our transcriptomic data may reflect the response of a tolerant subpopulation rather than the acute response of the general population.

### 2.7. Data Analysis

All data except the transcriptome data were analyzed with IBM SPSS 21.0 statistical software (SPSS Inc., Chicago, IL, USA). Graphs were generated using the GraphPad Prism 9.0 program (GraphPad Software Inc., La Jolla, CA, USA). The data are expressed as the mean ± standard error (SE).

Student’s *t* test was used to compare the secretion level, PBQ concentration, and antimicrobial activity of the oral secretions produced by RPW larvae subjected to external exposure to *M. anisopliae* or 0.1% Tween-80. The differences in the external immune defensive efficacy of oral secretions from RPW larvae at different time points after *M. anisopliae* infection and the corrected mortality of individuals at different concentrations of PBQ were analyzed via one-way analysis of variance (ANOVA), accompanied by Tukey’s honestly significant difference (HSD) test for multiple comparisons. The level of significance was set at α = 0.05 for all the statistical analyses.

Probit analysis was used to determine the toxicity regression equation, LC_10_, LC_50_ and the 95% confidence interval of PBQ in RPW larvae. This process involved converting the concentration-mortality response data into a log-probability model, where concentrations were transformed using the base 10.000 logarithm and mortality was transformed to corresponding probit values.

## 3. Results

### 3.1. Differences in the External Immune Responses of RPW Larvae to Pathogen Stress

#### 3.1.1. Changes in the Levels of Oral Secretions in Response to Pathogen Stress

**Figure 1:**
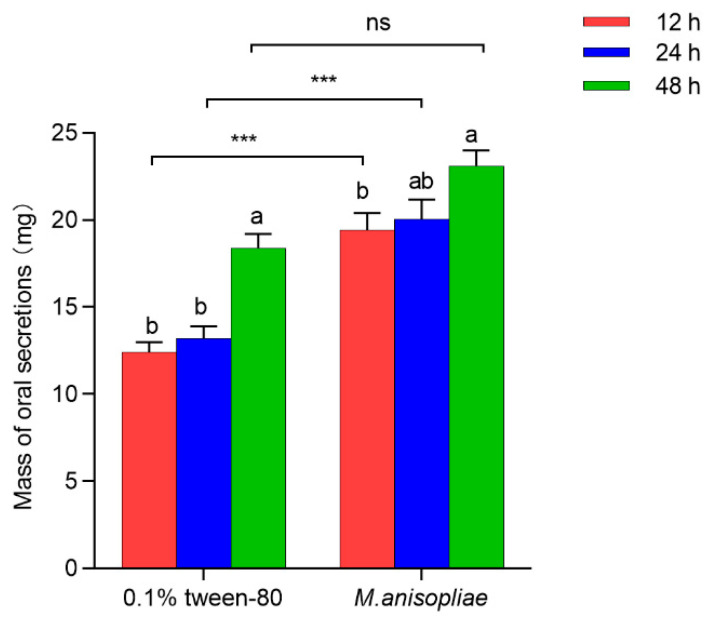
Levels of oral secretions released from third-instar red palm weevil (RPW) larvae treated with 0.1% Tween-80 or *M. anisopliae* at 12 h, 24 h, and 48 h postinfection in vitro. The graph shows the mean ± standard error. Bars for the same treatment labeled with different lowercase letters indicate statistically significant differences in the weight of oral secretions among different infection time points (one-way ANOVA followed by Tukey’s HSD multiple comparisons at *p* < 0.05). The asterisks marking Student’s *t* test results indicate that there is a significant difference between the two groups (***, *p* < 0.001), whereas “ns” indicates no significant difference (*p* > 0.05).

Compared with those in the 0.1% Tween-80 control group, the levels of oral secretions released from larvae at the third instar stage after external infection with *M. anisopliae* for 12 h (*t*_78_ = 6.119, *p* < 0.001) and 24 h (*t*_78_ = 5.202, *p* < 0.001) significantly increased by 0.56-fold and 0.52-fold, respectively; however, there was no significant difference at 48 h postinfection (*t*_78_ = 3.993, *p* = 0.683) (Figure 1). With increasing infection time, the secretory efficiency of the oral secretions of third-instar larvae significantly improved in both the 0.1% Tween-80 control group (*F*_2,117_ = 22.319, *p* < 0.001) and the *M. anisopliae* treatment group (*F*_2,117_ = 3.922, *p* = 0.022), which specifically showed that the weights of the oral secretions released by individuals after 48 h of infection were 0.48 times and 0.19 times greater than those after 12 h of infection (Figure 1). The results indicated that pathogens could significantly induce the production of oral secretions in RPW larvae when exposed externally. Within a certain stress response period, the longer the infection duration was, the stronger the ability to activate the host’s external immune system was, which led to increased levels of oral secretions, with the best induction effect observed at 24 h.

Further research revealed that 24 h after stress, the quantity of oral secretions from seventh-instar larvae was significantly greater than that from third-instar larvae (*t*_78_ = 9.477, *p* < 0.001), with average weights of 54.99 mg and 13.23 mg, respectively (Figure S1). This finding indicated that the level of oral secretions increased with the developmental stage of RPW larvae.

#### 3.1.2. The Effect of Pathogen Infection on the Concentration of PBQ in Oral Secretions

**Figure 2:**
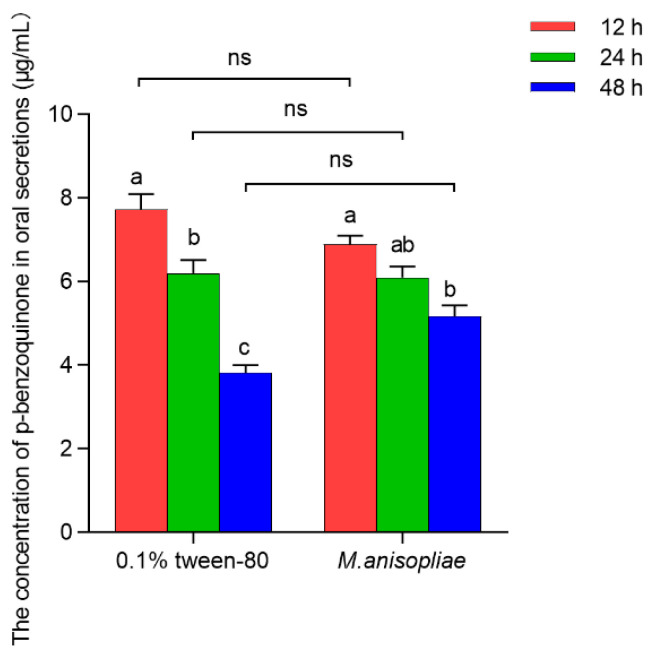
Amounts of p-Benzoquinone (PBQ) in oral secretions released from third-instar RPW larvae treated with 0.1% Tween-80 or *M. anisopliae* at 12 h, 24 h, and 48 h postinfection in vitro. The graph shows the mean ± standard error. Bars for the same treatment labeled with different lowercase letters indicate statistically significant differences in the concentration of PBQ among different infection time points (one-way ANOVA followed by Tukey’s HSD multiple comparisons at *p* < 0.05). The “ns” marking Student’s *t* test results indicate that there is no significant difference between the two groups (*p* > 0.05).

The concentration of PBQ in the oral secretions of third-instar larvae infected with *M. anisopliae* in vitro was not significantly different from that in the control larvae with 0.1% Tween-80 at 12 h (*t*_4_ = 2.022, *p* = 0.274), 24 h (*t*_4_ = 0.243, *p* = 0.629), or 48 h (*t*_4_ = 4.198, *p* = 0.658) (Figure 2). However, with increasing infection time, the concentration of PBQ in oral secretions of third-instar larvae significantly decreased in both the 0.1% Tween-80 control group (*F*_2,6_ = 43.907, *p* < 0.001) and the *M. anisopliae* treatment group (*F*_2,6_ = 12.324, *p* = 0.008), which specifically showed that the concentration of PBQ in oral secretions released by individuals after 48 h of infection was reduced by 50.65% and 24.98%, respectively, compared to that at 12 h postinfection (Figure 2). The results indicated that the longer the infection time of pathogens on RPW larvae in vitro was, the lower the concentration of PBQ as an external immune-active component in oral secretions was.

Further research revealed that 24 h after stress, there was no significant difference in the relative amount of PBQ in the oral secretions of third-instar larvae and seventh-instar larvae (*t*_4_ = 3.127, *p* = 0.904), with average concentrations of 6.20 μg/mL and 4.72 μg/mL, respectively (Figure S2). This finding indicated that the concentration of PBQ in oral secretions of RPW larvae was not significantly influenced by their instar stage.

#### 3.1.3. The Effect of Pathogen Infection on the Antimicrobial Efficacy of Oral Secretions

**Figure 3:**
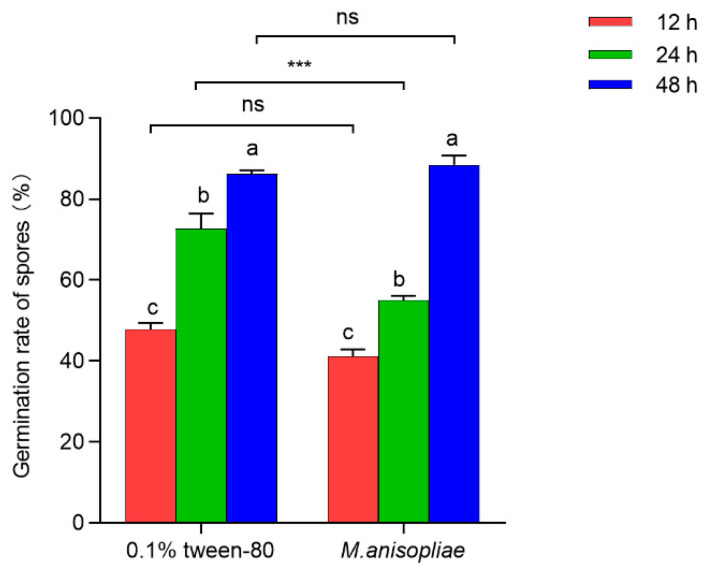
Antimicrobial efficacy of oral secretions released from third-instar RPW larvae treated with 0.1% Tween-80 or *M. anisopliae* at 12 h, 24 h, and 48 h postinfection in vitro. The graph shows the mean ± standard error. Bars for the same treatment labeled with different lowercase letters indicate statistically significant differences in the germination rate of *M. anisopliae* spores among different infection time points (one-way ANOVA followed by Tukey’s HSD multiple comparisons at *p* < 0.05). The asterisks marking Student’s *t* test results indicate that there is a significant difference between the two groups (***, *p* < 0.001), whereas “ns” indicates no significant difference (*p* > 0.05).

At different time points after third-instar larvae were infected with *M. anisopliae* in vitro, the oral secretions inhibited the germination of pathogenic spores to varying degrees (Figure S3). The antimicrobial activity of oral secretions from third-instar larvae externally exposed to *M. anisopliae* for 24 h was significantly greater than that of those exposed to 0.1% Tween-80 (*t*_10_ = 5.274, *p* = 0.001), and the germination rates of the spores were 55.03% and 72.74%, respectively (Figure 3). However, no significant differences were observed at 12 h (*t*_10_ = 2.787, *p* = 0.877) or 48 h (*t*_10_ = 0.907, *p* = 0.096) after exposure (Figure 3). Interestingly, the spore germination rate of *M. anisopliae* significantly increased by 32.61% for larvae exposed to 0.1% Tween-80 (*F*_2,14_ = 82.942, *p* < 0.001) and 47.40% for larvae exposed to *M. anisopliae* (*F*_2,16_ = 186.482, *p* < 0.001) from 12 h to 48 h postinfection (Figure 3), indicating the reduced antimicrobial activity of oral secretions. It can be seen that the oral secretions of RPW larvae 24 h after external infection by pathogens had the most significant inhibitory effect on the germination of their spores. Moreover, with the increased infection time of pathogens on RPW larvae in vitro, although the levels of the oral secretions increased, the concentration of PBQ as the main external immune-active component decreased, leading to a reduction in antimicrobial efficacy.

### 3.2. Toxic Effects of PBQ on RPW Larvae

#### 3.2.1. The Poisoning Symptoms of Larvae Exposed to PBQ

**Figure 4:**
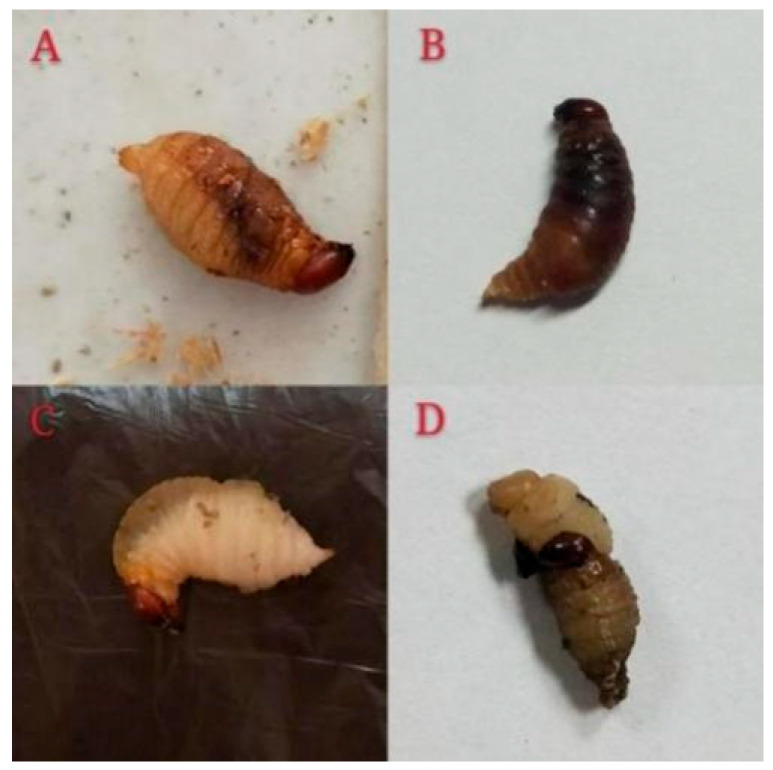
Poisoning symptoms of third-instar RPW larvae after exposure to PBQ. Photographs (**A**,**B**) show the phenomenon of cuticular melanization. Photographs (**C**,**D**) show the phenomenon of abnormal molting.

The third-instar larvae of RPW were unable to molt normally or exhibited varying degrees of cuticular melanization after exposure to PBQ, ultimately leading to individual death (Figure 4). We further speculated that the occurrence of these poisoning symptoms may be caused by the following two factors. On the one hand, PBQ inhibited the digestion and degradation of the old cuticle by affecting chitin metabolism, which resulted in an abnormal molting process. On the other hand, PBQ induced an immune defense response, activated phenoloxidase (PO), and then oxidized tyrosine to DOPA and dopamine, which resulted in the accumulation of melanin in the epidermis.

#### 3.2.2. Toxicity of PBQ to Larvae

**Figure 5:**
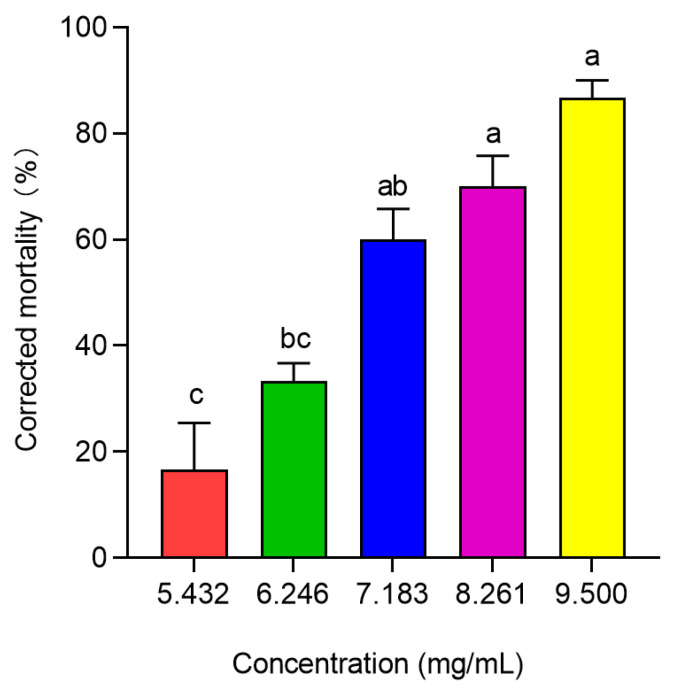
Corrected mortality of third-instar RPW larvae exposed to different concentrations of PBQ over a period of 10 d. The graph shows the mean ± standard error. Different lowercase letters indicate statistically significant differences in the corrected mortality among different concentrations of PBQ (one-way ANOVA followed by Tukey’s HSD multiple comparisons at *p* < 0.05).

The results of the bioassay showed significant differences in the toxicity of different concentrations of PBQ to third-instar RPW larvae (*F*_4,10_ = 23.833, *p* < 0.001; Figure 5). As the PBQ concentration increased, the corrected mortality of larvae within 10 d significantly increased from 16.67% to 86.67% (Figure 5).

After 12 h of treatment with PBQ, the toxic effects on the tested larvae were initially observed, and subsequently, a stable mortality of individuals was realized until 10 d. Within 10 d of feeding on sugarcane soaked in PBQ solution, the toxicity regression equation was fitted as *y* = −2.1134 + 8.4214*x*, with an LC_50_ of 6.982 × 10^3^ μg/mL (Table 1). However, the concentration of PBQ in the oral secretions of larvae infected with *M. anisopliae* in vitro for 24 h was 6.05 ± 0.28 μg/mL (Figure 2), which was far lower than the LC_10_ of 4.914 × 10^3^ μg/mL (Table 1), and PBQ decreased significantly with increasing total secretion levels (Figure 1 and Figure 2), indicating that the concentration of PBQ as an immune-active compound would not be toxic to the larvae themselves in the process of external immune defense.

### 3.3. Key Genes in RPW Larvae That Regulate the Toxication and Detoxification of the External Immune Factor PBQ

#### 3.3.1. Transcriptome Analysis

The total RNA extracted from the third-instar RPW larvae was of good quality, without obvious degradation or contamination (Figure S4). Additionally, the amount of each sample exceeded 1.5 μg, and the integrity of the RNA was high (Table S1), which met the requirements for transcriptome sequencing (RNA-seq).

After the completion of RNA-seq, the data were first subjected to quality control. The average sequencing depth was 30 million reads per sample, with mapping rates exceeding 85% to the reference transcriptome. Detailed per-sample metrics including raw read counts, Q scores, GC content, and mapping statistics were provided in Table S2. We found that the percentages of Q20 bases, Q30 bases and GC bases in the clean reads of each sample exceeded 96%, 90% and 43%, respectively (Table S2). These sequences presented a balanced base composition and high quality, which provided effective raw data for subsequent de novo assembly and accurate analysis.

The clean reads were then de novo assembled. The initial Trinity assembly generated 32,577 transcript sequences (hereafter referred to as “genes”). After clustering to remove redundancy, 4850 non-redundant transcripts (“unigenes”) were obtained, with an N50 of 1703 bp. The total number of assembled bases for all unigenes was 30,644,483, of which the maximum length was 47,430 bp, the minimum length was 201 bp, and the average length was 940 bp. The most abundant unigenes were less than 500 bp in length, followed by those ranging from 501 bp to 1000 bp (Figure S5). The obtained transcript sequences demonstrated high assembly completeness, allowing for subsequent gene functional annotation.

**Figure 6:**
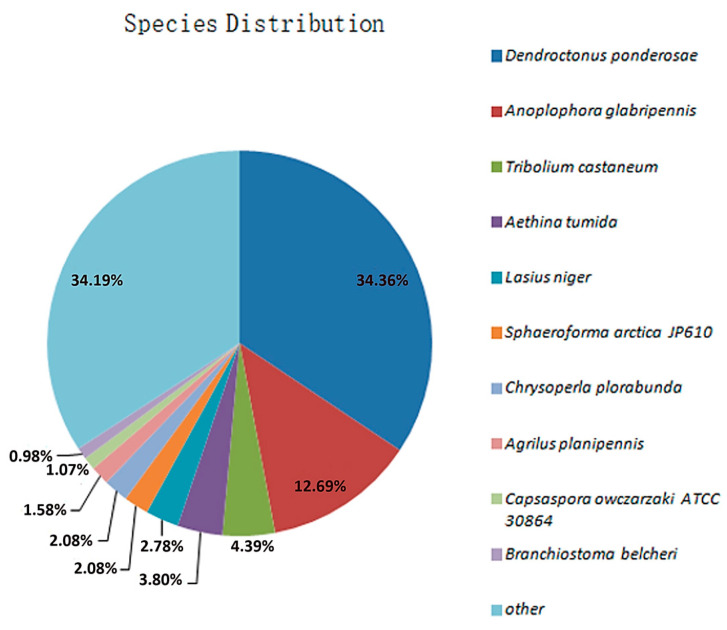
Distribution of species with genes homologous to those of the RPW after comparison with the Nr database.

A total of 19,397 unigenes were successfully annotated, accounting for 59.54% of the unigenes. Among these, the numbers of unigenes annotated using the Nr, KEGG, COG, and Swiss-Prot databases were 18,983 (58.27%), 15,983 (49.06%), 10,802 (33.16%), and 11,958 (36.71%), respectively (Table S3). A homology comparison with the Nr protein database revealed that the gene sequences in the transcriptome of RPW had the highest homology with some species from Coleoptera, sequentially, *Dendroctonus ponderosae*, *Anoplophora glabripennis*, and *Tribolium castaneum*, which aligned with 6522 genes (34.36%), 2408 genes (12.69%), and 834 genes (4.39%), respectively (Figure 6). These results indicated that the gene information for other species contained in the Nr database provided rich reference sequences for functional annotation of genes in this transcriptome, and it was speculated that the RPW shared a significant number of functionally similar genes with *D. ponderosae*.

**Figure 7:**
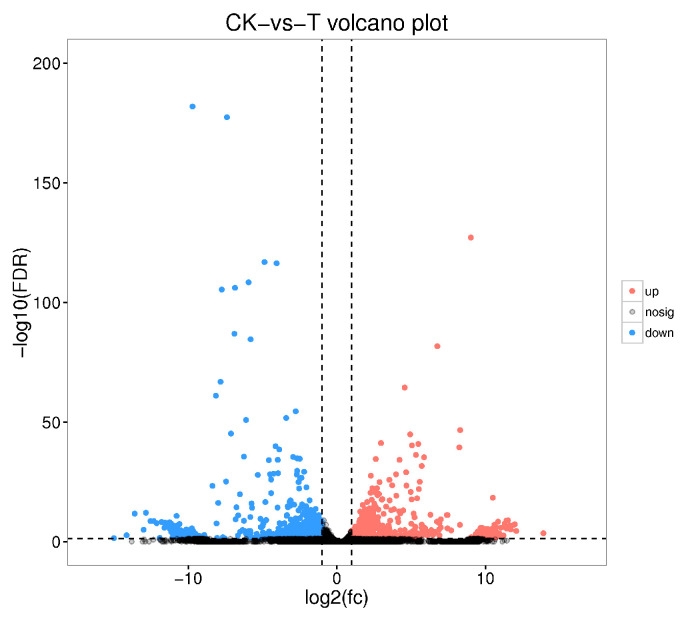
Volcano plot showing all differentially expressed genes in the transcriptome of third-instar RPW larvae between the distilled water control group and the PBQ treatment group. The red dots represent genes whose expression level was significantly up-regulated compared with that of the control. The blue dots represent genes whose expression level was significantly down-regulated compared with that of the control. The gray dots represent genes with expression levels that were not significantly different between the two groups.

Transcriptomic profiling of PBQ-tolerant larvae revealed gene expression patterns associated with tolerance. The expression levels of 1654 genes changed significantly after PBQ treatment, including 925 up-regulated genes and 729 down-regulated genes (Figure 7). A complete list of differentially expressed genes was provided in Table S4. The functional analysis of these differentially expressed genes revealed that these genes were involved in the stress response of third-instar RPW larvae to PBQ.

**Figure 8:**
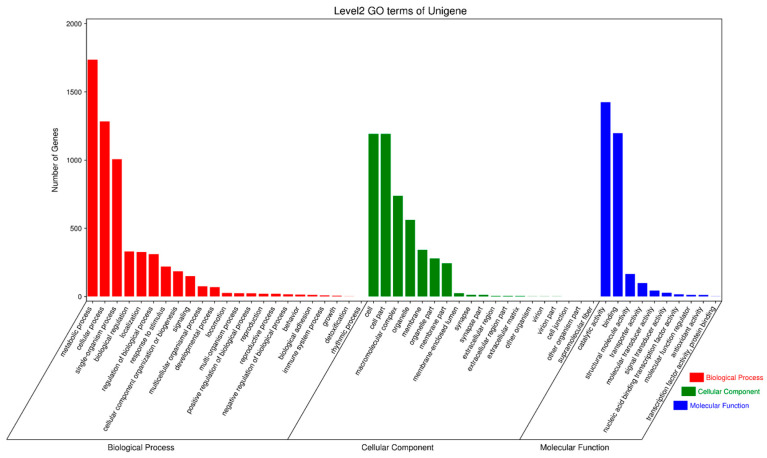
GO functional annotation of differentially expressed genes in the transcriptome of third-instar RPW larvae.

To further elucidate the biological behavior of the oxidative stress responses induced by the external immune-active component PBQ in the third-instar RPW larvae, the GO biological process, cellular component, and molecular function terms enriched in the differentially expressed genes were investigated. The biological processes most frequently enriched were the metabolic process (1737 genes), the cellular process (1283 genes), and the single-organism process (1007 genes); the most frequently enriched cellular components were the cell and the cell part, with 1192 genes each; and the molecular functions most frequently enriched were the catalytic activity and the binding, with 1424 and 1297 genes, respectively (Figure 8). These findings indicated that the RPW exhibited a strong metabolic response following treatment with PBQ.

**Figure 9:**
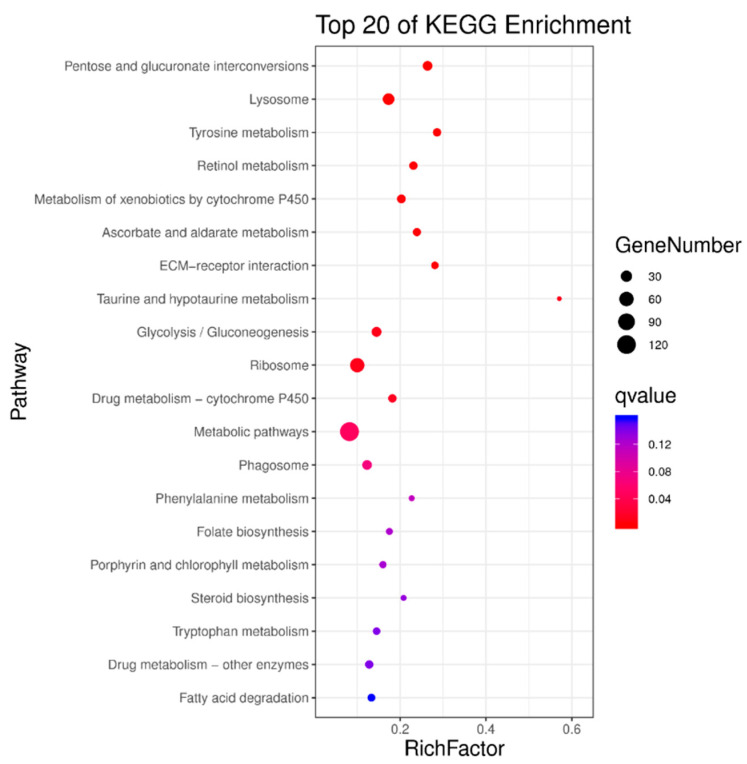
Enriched KEGG metabolic pathways for differentially expressed genes in the transcriptome of third-instar RPW larvae.

All differentially expressed genes were significantly enriched in one or more KEGG metabolic pathways, mainly including pentose and glucuronate interconversions, lysosome, tyrosine metabolism, retinol metabolism, metabolism of xenobiotics by cytochrome P450, ascorbate and aldarate metabolism, ECM-receptor interaction, taurine and hypotaurine metabolism, glycolysis/gluconeogenesis, ribosome, and drug metabolism-cytochrome P450 (Figure 9).

#### 3.3.2. Potential Mechanisms of Toxicity of PBQ in RPW Larvae

The third-instar RPW larvae exposed to PBQ failed to molt normally or exhibited varying degrees of cuticular melanization, ultimately resulting in death (Figure 4). Each growth and development stage of insects is accompanied by shedding of the old epidermis and the formation of a new epidermis, which are closely related to chitin. Chitin and tanned protein increase the rigidity of the epidermis, and chitinase (CHI) is found in the secretion of the molting gland. Therefore, the synthesis and degradation of chitin in insect cuticles exhibit a dynamic equilibrium. By analyzing the expression differences in related genes in the transcriptome, we identified three up-regulated genes and two down-regulated genes from the CHI family (Table 2), suggesting that PBQ affects chitin metabolism, thereby potentially inhibiting the digestion and degradation of the old cuticle. Furthermore, the up-regulation of three genes from the PO family (Table 2) indicated that the expression level of PO was significantly increased by the induction of PBQ, after which tyrosine was catalytically oxidized to DOPA and dopamine to finally form melanin.

#### 3.3.3. Potential Metabolic Detoxification Mechanisms of RPW Larvae Against PBQ

After the third-instar RPW larvae were treated with PBQ, the expression levels of three genes in the cytochrome P450 (CYP450) family were up-regulated, and those of two genes were down-regulated; moreover, the expression levels of two genes in the glutathione S-transferase (GST) family and seven genes in the ATP-binding cassette transporter (ABC) C subfamily were up-regulated (Table 3). These results further indicated that multiple genes encoding detoxifying enzymes, including *CYP450*, *GST*, and *ABC*, exhibited significant up-regulation in response to stimulation with PBQ. This suggests their potential involvement in the metabolism and detoxification of PBQ by RPW larvae.

## 4. Discussion

External immune defensive behaviors endow insects with the ability to thrive in complex and changeable ecological environments. Quinones and other chemical components in the external secretions of many Coleoptera insects have been shown to inhibit the growth of microorganisms [39]. This study highlights that the external immune defense of the RPW can be induced by pathogens and that the induction efficiency is relatively high. Specifically, the level of oral secretions from third-instar larvae significantly increased after infection with *M. anisopliae* in vitro. This result is consistent with the findings of He et al. [31] in soldier ants of *M. darwiniensis*. Therefore, when these insects are subjected to pathogen stress, their external immune responses may be enhanced by the increased release of oral secretions. Surprisingly, we found that both the concentration of PBQ in oral secretions and the antimicrobial efficacy of these secretions decreased with increasing duration of infection. Furthermore, the levels of oral secretions from seventh-instar larvae infected with *M. anisopliae* for 24 h were significantly greater than those of third-instar larvae, indicating that the release of external immune defensive secretions was influenced by their developmental stage.

However, benzoquinones are toxic and can have adverse physiological effects on the secretors, potentially leading to death [39]. Therefore, the concentration of PBQ in the external secretions of insects must be effectively regulated by the secretors themselves. The biosynthesis of quinones depends on the availability of tyrosine, which also serves as the precursor for the PO cascade [40,41]. The PO cascade, which is a central enzymatic cascade in the immune system of arthropods, also participates in the melanization and hardening of the cuticle [42]. On the one hand, Pu et al. [10] identified three *ARSB* genes associated with the biosynthesis of PBQ in oral secretions from RPW larvae infected with *M. anisopliae* in vitro. On the other hand, our research observed that, after exposure to PBQ, RPW larvae presented varying degrees of cuticular melanization. The integration of these two points further allows us to propose a hypothetical model of a trade-off relationship between the biosynthesis of PBQ and the PO cascade reaction, shifting from quinone synthesis to cuticle melanization and sclerotization. By reasonably controlling the concentration of PBQ in external secretions through this trade-off, it is possible to increase the fitness of insects in pathogenic environments.

Under normal circumstances, the concentration of PBQ in the external secretions of RPW larvae does not have toxic effects on individuals. Notably, the concentration of PBQ used in our toxicity assays was approximately three orders of magnitude greater than the physiological concentration found in larval oral secretions. This difference highlights the effective regulatory mechanisms that maintain PBQ below toxic levels during normal immune defense. The high concentration used in our experiments represents an acute exposure scenario designed to elicit measurable phenotypic and transcriptomic responses, which may not reflect the physiological conditions but rather reveal the potential detoxification mechanisms when the regulatory system is overwhelmed. Although PBQ resulted in the highest corrected mortality of 86.67% compared with that of third-instar RPW larvae, with an LC_50_ of 6.982 × 10^3^ μg/mL, the average concentration of PBQ in oral secretions of 6.05 μg/mL was significantly lower than the LC_10_ of 4.914 × 10^3^ μg/mL for individuals. It should be noted, however, that this direct concentration comparison requires careful interpretation because of differences in exposure routes and bioavailability. In our toxicity assay, larvae were continuously exposed to PBQ via oral ingestion from soaked sugarcane, which may result in higher systemic bioavailability than the localized and transient presence of PBQ in oral secretions during natural defense responses. Therefore, the actual toxicological risk under ecological conditions may be overestimated if it is based only on concentration equivalence and does not consider exposure dynamics.

We also noted that the secretion of PBQ had a threshold, corroborating the conjecture of Gokhale et al. [26], as the concentration of PBQ does not necessarily change with varying levels of secretion. These results can be explained by the trade-off between quinone synthesis (external immune defense) and the PO cascade (internal immune defense) [43]. Upon sensing pathogen stress, the epidermis of insects become hardened and tanned [44], leading to the secretion of external defensive compounds primarily composed of PBQ as the main active component. The hardening and tanning of the epidermis not only provide resistance against pathogen infection in vitro but also prevent the toxicity caused by PBQ to the host itself. When a pathogen invades the insect body, there is a shift from quinone synthesis to the PO cascade to precisely regulate the concentration of PBQ in the secretions. This ensures that the secretory level of PBQ is sufficient to exert external immune efficacy while avoiding harm to the secreting organism.

The transcriptomic profile obtained represents survivors of PBQ exposure, which may have activated specific detoxification mechanisms not present in the general population. PO is a key enzyme in the synthesis of melanin that is capable of oxidizing tyrosine into DOPA and dopamine [45]. In insects, PO primarily exists in tissues such as the serum, hemocytes, epidermis, and midgut [45]. Research has shown that the melanization of *Anopheles gambiae* is determined by six prophenoloxidase (*PPO*) genes [46]. The expression level of *PPO* is often regulated at both the translation and post-translation stages, and PPO can activate PO [47]. Similarly, the expression levels of three PO genes were significantly up-regulated in RPW larvae after exposure to PBQ. We hypothesize that PBQ exposure was associated with up-regulation of PO genes, which may lead to increased melanin production, resulting in varying degrees of cuticular darkening and ultimately individual death. However, this hypothesized causal chain requires direct experimental validation in future work.

The insect epidermis is composed of chitin, and the synthesis and degradation of chitin cooperate to facilitate the shedding of the old epidermis and the formation of a new epidermis during larval development [48]. CHI is widely present in insects and is a member of a class of glucosamine hydrolases with low molecular weights that can degrade the old epidermis and peritrophic membrane during insect molting [48,49,50]. A previous study indicated that *T. castaneum* died after interference with the CHI family genes *TcChi5*, *TcChi10*, and *TcChi7* [51]. In our study, five CHI genes in the larvae of RPW were significantly induced by PBQ, which disrupted the balance of chitin metabolism, inhibited the normal molting process, and ultimately led to death. These results suggest that PBQ can affect the digestion and degradation of the old cuticle by RPW larvae, with the CHI genes possibly playing an important role.

Previous studies have demonstrated that the metabolic detoxification process in insects can be categorized into three stages on the basis of the types and functions of the detoxifying enzymes involved. In stage I, the functional groups introduced or released by CYP450 and carboxylesterase (CarE) directly act on the surface of toxin molecules, making the toxin molecules more hydrophilic. In stage II, GST and UDP glycosyltransferase form conjugates with toxin molecules by methylation, acetylation, phosphorylation, and sulfonation, thereby increasing their water solubility and preventing their diffusion through membranes. Stage III involves the efflux of toxin molecules through the transmembrane transport function of ABC transporters [52,53]. A crucial metabolic enzyme system involved in the metabolic detoxification of insects is CYP450, which can metabolize various endogenous and exogenous compounds [54]. Another important detoxifying enzyme in insects is GST, which catalyzes conjugation reactions between reduced glutathione (GSH) and electrophilic groups of toxic substances, facilitating their efflux in a non-enzymatic form [52,53,55]. The activity of GST and the content of CYP450 in *Spodoptera exigua* initially increased after treatment with methoxyfenozide, indicating a physiological and biochemical stress response of the insect to methoxyfenozide, which promotes its metabolic processes [55,56]. Furthermore, most ABC transporters exhibit transport activity and transport metabolites and other substrates across the membrane in a reverse concentration gradient using the energy derived from ATP hydrolysis [57]. Multidrug resistance-associated protein (MRP) belongs to the ABC transporter C subfamily. When *Plutella xylostella* was treated with chlorantraniliprole at the LC_50_, *ABCC3*, *ABCC4*, and *ABCC5* were overexpressed, indicating that these three ABC family genes exhibit strong stress responses to chlorantraniliprole and may be involved in the detoxification metabolism of chlorantraniliprole in *P. xylostella* [58]. In the present study, we found that PBQ significantly up-regulated 3 genes encoding *CYP450*, 2 genes encoding *GST*, and 7 genes encoding *ABC* in RPW larvae, whereas only 2 genes encoding *CYP450* were significantly down-regulated. This result is similar to results observed in many insects that can be induced by exogenous compounds to express the *CYP450*, *CarE*, and *GST* genes [59]. Notably, the transcriptomic profile obtained represents survivors of PBQ exposure, which may have activated specific detoxification mechanisms not present in the general population. Therefore, this pattern of gene expression is consistent with a coordinated detoxification response and provides a foundation for future functional studies to validate the roles of these candidate genes in conferring tolerance.

Several limitations of this study should be noted. While we identified candidate genes involved in detoxification and stress response, functional validation through qPCR of key DEGs and enzymatic assays will be necessary to confirm their specific roles. Future studies should also explore dose–response relationships at concentrations closer to physiological levels.

## 5. Conclusions

In summary, PBQ, an external immune-active compound derived from oral secretions, can cause excessive immune responses and the overproduction of melanin in the host by correlating with up-regulating PO gene expression, resulting in cuticular melanization and the death of RPW larvae. Additionally, this compound may disrupt the balance of chitin metabolism by regulating CHI gene expression, causing abnormal molting in RPW larvae and their death. However, the coordinated upregulation of several genes encoding detoxifying enzymes, such as *CYP450*, *GST*, and *ABC*, suggests a potential mechanism for mitigating PBQ toxicity in RPW larvae. These findings provide crucial candidate genes and pathways for further elucidating the underlying molecular mechanisms behind the tolerance of RPW to the external immune factor PBQ.

## Figures and Tables

**Table 1:** Regression analysis of the toxicity of PBQ against third-instar RPW larvae for 10 d.

LC_10_ (95% Confidence Interval) (μg/mL)	LC_50_ (95% Confidence Interval) (μg/mL)	Slope (±SE)	*χ*^2^ (*df*)	Coefficient of Determination
4.914 × 10^3^ (4.044 × 10^3^–5.462 × 10^3^)	6.982 × 10^3^ (6.532 × 10^3^–7.427 × 10^3^)	8.402 (±1.405)	6.473 (13)	0.9936

**Table 2:** Differential expressions of chitinase and phenoloxidase genes in the third-instar RPW larvae exposed to PBQ.

Family	Homologous Genes	Species	log_2_ (FC)	Variations	GeneBank ID
CHI	CHI 3 isoform X2	*Tribolium castaneum*	6.300	Up	Unigene0013264
	CHI	*Sitophilus oryzae*	−1.388	Down	Unigene0009970
	CHI -like protein Idgf4 isoform X1	*Dendroctonus ponderosae*	1.457	Up	Unigene0025235
	CHI -like protein Idgf4	*Dendroctonus ponderosae*	1.085	Up	Unigene0021576
	CHI	*Gregarina niphandrodes*	−2.332	Down	Unigene0011156
PO	PO 2	*Dendroctonus ponderosae*	2.655	Up	Unigene0030095
	PO 2	*Dendroctonus ponderosae*	2.385	Up	Unigene0022841
	PO 2	*Dendroctonus ponderosae*	1.037	Up	Unigene0031065

**Table 3:** Differential expressions of related genes of detoxifying enzymes in the third-instar RPW larvae exposed to PBQ.

Family	Homologous Genes	Species	log_2_ (FC)	Variations	GeneBank ID
CYP450	CYP450 CYP6CR2	*Dendroctonus ponderosae*	−2.282	Down	Unigene0007108
	CYP450 CYP6BX1	*Dendroctonus ponderosae*	1.147	Up	Unigene0023500
	CYP450 CYP6BX1	*Dendroctonus ponderosae*	−2.450	Down	Unigene0031050
	CYP450 307a1-like	*Dendroctonus ponderosae*	3.421	Up	Unigene0020614
	CYP450 6a13	*Dendroctonus ponderosae*	1.019	Up	Unigene0026373
GST	GST1-like isoform X2	*Dendroctonus ponderosae*	1.593	Up	Unigene0009075
	GST, partial	*Rhynchophorus ferrugineus*	3.973	Up	Unigene0022356
ABC	MRP lethal(2)03659 isoform X1	*Dendroctonus ponderosae*	1.025	Up	Unigene0012676
	MRP lethal(2)03659	*Dendroctonus ponderosae*	2.966	Up	Unigene0029437
	MRP lethal(2)03659	*Dendroctonus ponderosae*	2.493	Up	Unigene0021880
	MRP lethal(2)03659	*Dendroctonus ponderosae*	4.293	Up	Unigene0029438
	MRP lethal(2)03659	*Dendroctonus ponderosae*	1.168	Up	Unigene0027106
	MRP 4-like	*Dendroctonus ponderosae*	1.017	Up	Unigene0004482
	MRP 4-like	*Dendroctonus ponderosae*	1.263	Up	Unigene0004481

## Data Availability

The data are available in a publicly accessible repository. The original data presented in this study are openly available in FigShare at https://doi.org/10.6084/m9.figshare.30006430. The raw RNA-seq sequencing data have been deposited in the NCBI Sequence Read Archive (SRA) under the BioProject accession number PRJNA1333853.

## Supplementary Materials

The following supporting information can be downloaded at: https://www.mdpi.com/article/10.3390/insects16101044/s1, Figure S1: Levels of oral secretions released from third-instar and seventh-instar RPW larvae subjected to stress for 24 h. Figure S2: Amounts of PBQ in oral secretions released from third-instar and seventh-instar RPW larvae subjected to stress for 24 h. Figure S3: Optical microscopy images of *M. anisopliae* spores exposed to oral secretions from third-instar RPW larvae. Figure S4: Agarose (1%) gel electrophoresis diagram of total RNA extracted from third-instar RPW larvae. Figure S5: Length distribution of unigenes assembled by transcriptome sequencing for third-instar RPW larvae. Table S1: Concentrations and integrity of total RNA extracted from third-instar RPW larvae. Table S2: Quality control statistical analysis of the transcriptome data for third-instar RPW larvae. Table S3: Annotation of genes from third-instar RPW larvae by four major nucleotide and protein databases. Table S4: Complete list of DEGs in the third-instar RPW larvae exposed to PBQ.
