# Supplementary material for: Comparative Transcriptome Analysis of Rhynchophorus ferrugineus (Coleoptera: Curculionidae) Reveals Potential Mechanisms Involved in the Toxication and Detoxification of the External Immune Compound p-Benzoquinone Present in Oral Secretions
Source: Insects. 2025 Oct 11;16(10):1044. doi: 10.3390/insects16101044 (PMC12564192; doi:10.3390/insects16101044)
Supplement: Supplementary file 1 [file insects-16-01044-s001.zip › Figures S1-S5, Tables S1-S3.pdf]

Supplementary materials

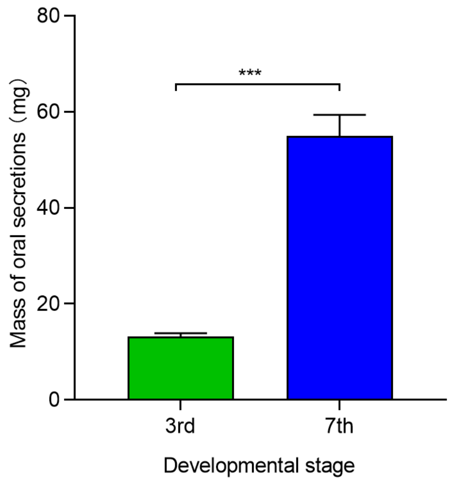

Figure S1 Levels of oral secretions released from third-instar and seventh-instar red palm weevil larvae subjected to stress for 24 h. The graph shows the mean ± standard error. The asterisks marking Student's *t* test results indicate that there is a significant difference in the weight of oral secretions between the third-instar larvae and the seventh-instar larvae (***, $p<0.001$).

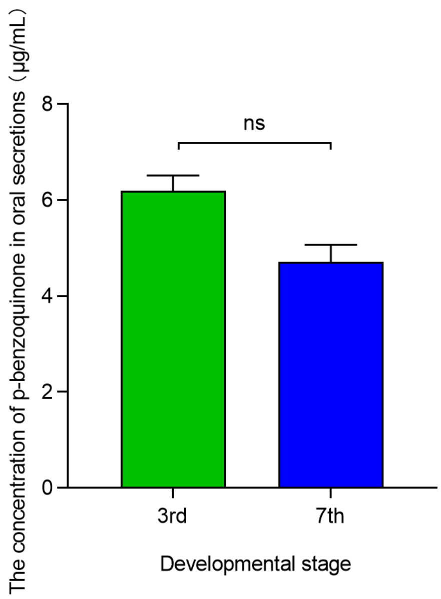

Figure S2 Amounts of p-benzoquinone in oral secretions released from third-instar and seventh-instar red palm weevil larvae subjected to stress for 24 h. The graph shows the mean ± standard error. The "ns" marking Student's *t* test result indicates that there is no significant difference in the concentration of p-benzoquinone between the third-instar larvae and the seventh-instar larvae (p>0.05).

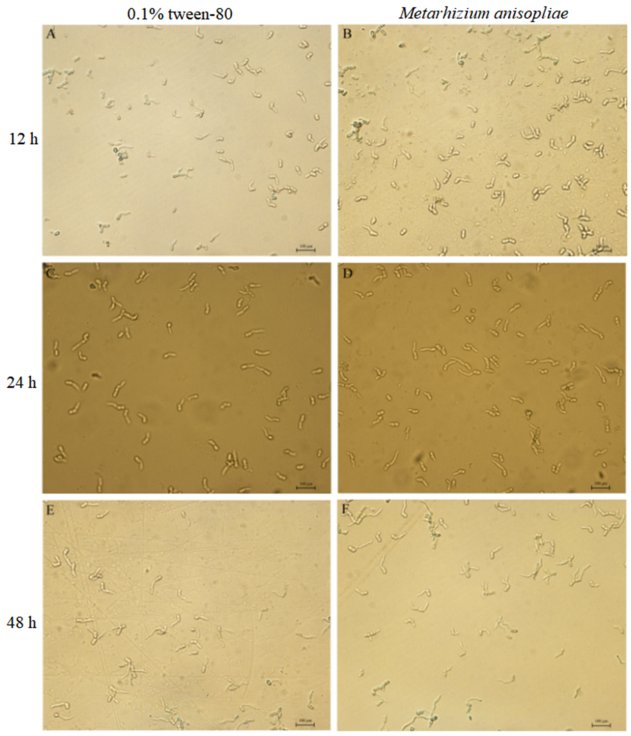

Figure S3 Optical microscopy images of *Metarhizium anisopliae* spores exposed to oral secretions from third-instar red palm weevil larvae. The scale bar represents 100 μm.

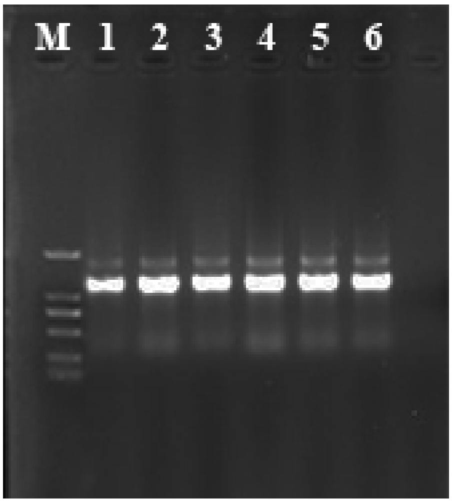

Figure S4 Agarose (1%) gel electrophoresis diagram of total RNA extracted from third-instar red palm weevil larvae. The label “M” shows the DL2000 DNA marker, the bands of which represent 2000 bp, 1000 bp, 750 bp, 500 bp, 250 bp and 100 bp from top to bottom. The labels “1”, “2” and “3” represent three biological replicates for the control group receiving distilled water. The labels “4”, “5” and “6” represent three biological replicates for the treatment group receiving p-benzoquinone.

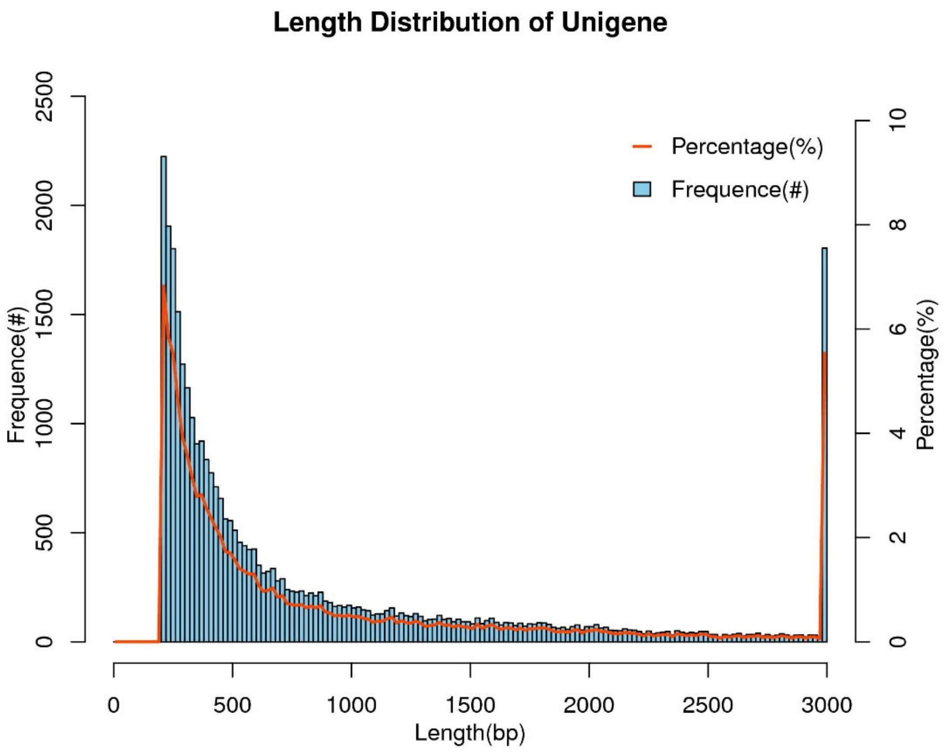

Figure S5 Length distribution of unigenes assembled by transcriptome sequencing for third-instar red palm weevil larvae. The x-axis represents the length of the assembled unigenes, whereas the y-axis represents the number of unigenes of the corresponding length.

Table S1 Concentration and integrity of total RNA extracted from the third-instar red palm weevil larvae

Sample number	Sample name	Concentration (ng/μL)	Volume (μL)	Total amount (μg)	$OD_{260/280}$	$OD_{260/230}$	28S/18S
1	CK-1	256	35	8.96	2.04	2.18	0.1
2	CK-2	326	36	11.74	1.96	2.17	0.0
3	CK-3	298	37	11.03	2.02	1.74	0.1
4	T-1	409	37	15.13	1.92	1.8	0.0
5	T-2	338	36	12.17	1.96	1.55	0.0
6	T-3	319	36	11.48	2	2.05	0.1

Note: CK: The control group of distilled water; T: The treatment group of p-benzoquinone.

Table S2 Quality control statistics of transcriptome data for the third-instar red palm weevil larvae

Sample	The number of clean reads	The number of clean bases	The ratio of Q20 bases	The ratio of Q30 bases	The ratio of GC bases	Raw reads	Mapping rate (%)	Clean rate (%)
CK-1	48498108	7238106339	98.53	95.32	45.48	48544386	85.04	99.90
CK-2	41575324	6189100718	96.55	90.84	45.45	41625798	84.64	99.88
CK-3	38315348	5712793968	97.00	91.82	45.07	38357560	84.34	99.89
T-1	40753694	6073253446	96.59	90.97	43.97	40793202	85.46	99.90
T-2	39134830	5846642337	96.92	91.71	45.38	39175710	85.48	99.90
T-3	43383026	6476219851	98.43	95.14	45.68	43425550	86.53	99.90

Note: CK: The control group of distilled water; T: The treatment group of p-benzoquinone.

Table S3 Annotation of genes from the third-instar red palm weevil larvae in four major nucleotide and protein databases

Database	Unigene number	Percentage (%)
Annotated in Nr	18983	58.27
Annotated in KEGG	15983	49.06
Annotated in COG	10802	33.16
Annotated in Swiss-Prot	11958	36.71
Annotated genes	19397	59.54
Without annotated genes	13180	40.46
Total Unigenes	32577	100
